# Lack of relationship between TIMP-1 tumour cell immunoreactivity, treatment efficacy and prognosis in patients with advanced epithelial ovarian cancer

**DOI:** 10.1186/1471-2407-10-185

**Published:** 2010-05-07

**Authors:** Karina Dahl Steffensen, Marianne Waldstrøm, Rikke Kølby Christensen, Annette Bartels, Nils Brünner, Anders Jakobsen

**Affiliations:** 1 Department of Oncology, Vejle Hospital, Kabbeltoft 25, DK-7100 Vejle, Denmark; 2 Department of Pathology, Vejle Hospital, Kabbeltoft 25, DK-7100 Vejle, Denmark; 3 Department of Veterinary Disease Biology/Section for Pathobiology, Faculty of Life Sciences, University of Copenhagen, Ridebanevej 3, DK-1870 Frederiksberg C, Denmark; 4 Institute of Regional Health Services Research, University of Southern Denmark, Winsløwparken 25-3, DK-5000 Odense C, Denmark

## Abstract

**Background:**

Tissue inhibitor of metalloproteinase 1 (TIMP-1) is a natural inhibitor of the matrix metalloproteinases (MMPs) which are proteolytic enzymes involved in degradation of extracellular matrix thereby favoring tumour cell invasion and metastasis. TIMP-1 activity in tumour tissue may therefore play an essential role in the progression of a malignant tumour.

The primary aim of the present study was to evaluate TIMP-1 protein immunoreactivity in tissue from primary ovarian cancer patients and associate these findings with the course of the disease including response to treatment in the individual patient.

**Methods:**

TIMP-1 was assessed by immunohistochemistry (in tissue micro arrays) in a total of 163 ovarian cancer specimens obtained from primary debulking surgery during 1991-1994 as part of a randomized clinical protocol.

**Results:**

Positive TIMP-1 immunoreactivity was found in 12.3% of the tumours. The median survival time for the 143 patients with TIMP-1 negative tumours was 23.7 months [19.0-29.4] 95% CI, while the median survival time for the 20 patients with TIMP-1 positive tumours was 15.9 months [12.3-27.4] 95% CI. Although a difference of 7.8 months in median overall survival in favor of the TIMP-1 tumour negative patients was found, this difference did not reach statistical significance (*p *= 0.28, Kaplan-Meier, log-rank test). Moreover, TIMP-1 immunoreactivity was not associated with CA125 response (p = 0.53) or response at second look surgery (p = 0.72).

**Conclusion:**

TIMP-1 immunoreactivity in tumour tissue from patients with primary epithelial ovarian cancer did not correlate with patient survival or response to combination platinum/cyclophosphamide therapy.

## Background

Epithelial ovarian cancer is the leading cause of gynecologic-related cancer deaths. The cornerstone of treatment is debulking surgery and platinum-based chemotherapy with succeeding complete clinical response for the majority of the patients. Unfortunately, a high percentage of the patients experience relapse of their disease, and treatment for recurrent disease has not achieved convincing success rates. Therefore, scientific efforts have focused on improving current treatment. In this context, the use of biological or molecular markers for predicting patient outcome including selection of the most effective treatment for the individual patient has attracted a lot of interest. The most widely used tumour marker in ovarian cancer is CA125. This biomarker is used to evaluate diagnosis, treatment efficacy and to monitor disease status although there are several limitations in the interpretation of CA125 such as inadequate sensitivity and specificity. In recent years, a new biological marker of potential usefulness for early detection, prognosis, prediction and monitoring of a variety of tumour types has been suggested [1]. This marker, tissue inhibitor of matrix metalloproteinase-1 (TIMP-1) is one among four members of the TIMP family. These proteins are natural inhibitors of the matrix metalloproteinases (MMPs) and play a key role in maintaining homeostasis of the extracellular matrix by controlling the proteolytic activity of the MMPs. In addition to its role in regulating MMPs, TIMP-1 has also been shown to stimulate cell proliferation, exhibit anti-apoptotic effects and to influence angiogenesis [1-6].

TIMP-1 immunohistochemical studies have shown increased TIMP-1 immunoreactivity in gastric, breast and renal carcinomas [7-9]. Moreover, several studies have found a correlation between TIMP-1 protein levels in blood or tumour tissue obtained from various cancer types (breast, esophageal, gastric, renal, colorectal and non-small cell lung carcinoma) and prognosis [7,8,10-15]. More recently, TIMP-1 levels have been associated with response to therapy of breast [9,16] and colorectal cancer [10]. Over-expression of TIMP-1 has also been reported in ovarian cancer patients compared to patients with borderline tumours, benign ovarian tumours or with normal ovaries. The majority of these ovarian cancer studies have investigated serum [17,18] or plasma [19] TIMP-1 levels while only a few have investigated tissue TIMP-1 concentration by ELISA technique [20], tissue TIMP-1 by in situ mRNA hybridization and/or immunohistochemistry [21] or TIMP activity by reverse zymography [22] and all found increased TIMP-1 levels/expression in ovarian cancer specimens. The assessment of serum concentrations of TIMP-1 (and some metalloproteinases) appeared to be useful in differentiating between malignant, borderline and benign ovarian tumours [17,18] although some overlaps between the groups were apparent. Furthermore, the studies were under-powered for diagnostic purposes and no sensitivity or specificity was reported.

Only very limited published data exist on the prognostic value of TIMP1 in ovarian cancer. One study including 40 patients with ovarian cancer found that increasing preoperative plasma levels of TIMP-1 were associated with poor survival [19]. A second study found that high preoperative serum concentrations of TIMP-1 from 59 ovarian cancer patients were correlated with decreased recurrence-free survival and overall survival in univariate analysis [18]. However, no prior studies have investigated the association between TIMP-1 immunoreactivity and patient prognosis in epithelial ovarian cancer patients.

The primary aim of the present study was therefore to evaluate whether TIMP-1 immunoreactivity in tissue from primary ovarian cancer patients was associated with overall survival of the patients. Moreover, it was tested whether TIMP-1 immunoreactivity was associated with treatment efficacy as evaluated by CA125 response and response at second look surgery following treatment with carboplatin and cyclophosphamide.

## Methods

### Study Population

From 1991 to 1994, 222 patients were prospectively enrolled in the DACOVA (Danish Ovarian Cancer Study Group) 9101 protocol [23]. In this study patients were randomized to receive combination chemotherapy with carboplatin and cyclophosphamide (500 mg/m2) with carboplatin at a dose of AUC4 in one treatment arm or at a dose of AUC8 in the other treatment arm. Neither preliminary nor long-term results [24] showed any effect of the higher carboplatin dose. Patients with stage II-IV and with confirmed epithelial ovarian cancer were included and collected data were entered into case report forms. The protocol was approved by The Danish Biomedical Research Ethics Committee and all patients were required to provide written informed consent prior to inclusion.

All patients were included into the protocol during 1991-1994. At that time the RECIST criteria did not exist and therefore, the international recognized GCIG CA-125 response criteria and not RECIST criteria were used for response evaluation. Moreover, in ovarian cancer, there are often no reliably measurable target lesions due to predominantly diffuse peritoneal disease. Hence, serum CA-125 levels are extensively used to assess response and define progression in ovarian cancer and often mirror the RECIST/WHO criteria [25].

CA125 serum levels were analyzed by routine methods at the Clinical Chemistry Departments of the treating hospitals. Full CA125 data were available on 106 of the patients. The study was carried out in compliance with the Helsinki Declaration and the Danish Biomedical Research Ethics Committee approved the study according to Danish law.

### Preparation of tissue microarrays

Formalin-fixed, paraffin-embedded tissue blocks from the primary tumour debulking, prior to first line chemotherapy, were retrieved from the Regional Departments of Pathology. All cases underwent central pathology review and were classified according to the WHO histological classification of ovarian tumours and graded according to Silverberg [26]. Adequate specimens from 163 patients were selected for analysis. Patients not included in the present study were a result of missing blocks, lack of patient data, wrong pathology numbers or lack of residual tumour tissue in the collected blocks. Furthermore, at the central pathology review nine patients were considered as not having primary ovarian cancer but instead six cases with metastatic disease from another primary tumour and three cases with borderline ovarian tumours.

**Figure 1:**
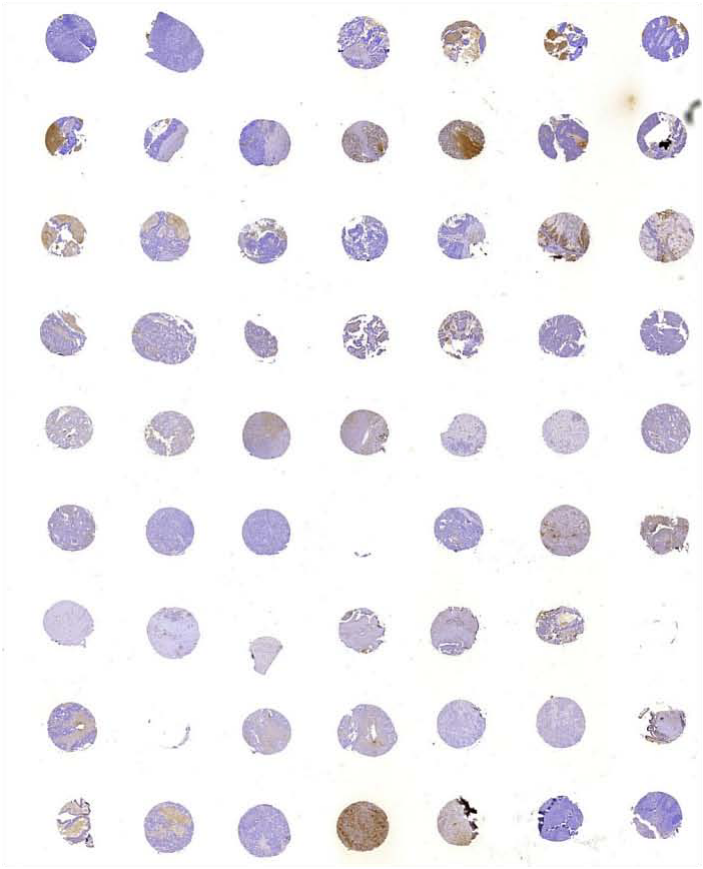
TMA used for TIMP-1 immunohistochemistry

Tissue microarray (TMA) using Advanced Tissue Arrayer ATA-100 (Chemicon, AH Diagnostics, Aarhus, Denmark) was constructed from 163 patients. Specimens were retrieved from areas of the original donor paraffin block selected by a pathologist and arrayed into a newly constructed recipient block. Tissue cores were 1 mm in diameter (Fig 1). For each tumour 2 or 3 core samples of tumour tissue were acquired from the donor block. The TMA block also contained samples of normal tonsillar and appendix tissue (placed in specific corners of the TMA block to ensure correct orientation when examining the slides), which served as internal negative control. A slide with breast cancer tissue served as internal positive controls.

For 20 patients whole-tissue sections were also evaluated for TIMP-1 staining to investigate possible heterogeneity in the TIMP-1 immunoreactivity and to investigate the correlation to the staining observed in the corresponding TMA's.

### TIMP-1 immunohistochemical staining

Freshly cut TMA and whole-slide paraffin sections were stained with the monoclonal antibody VT-7, raised against recombinant human TIMP-1 [27]. The antibody is of the IgG_1 _subtype and was used in the concentration 0.25 μg/ml.

As a negative control, the irrelevant IgG_1 _monoclonal antibody raised against trinitrophenyl hapten (TNP) was used. For each immunohistochemical (IHC) run, a positive control case known to contain TIMP-1 was included. Reagents used for IHC were obtained from Dako (Dako Denmark A/S, Glostrup, Denmark) and used according to the manufacturer's instructions. All staining procedures were performed manually.

The paraffin sections (3 μm) were de-waxed in xylene and rehydrated through a graded series of ethanol. Antigen retrieval was carried out by boiling the sections for 10 min. in a conventional microwave oven in 10 mM citrate buffer pH 6.0, followed by 30 min in the hot buffer at room temperature. To block endogenous peroxidase activity the sections were treated with 1% hydrogen peroxide for 10 min. Sections were incubated with primary antibody overnight at 4°C.

Bound primary antibody was detected with Advance HRP (Code No. K4068, Dako Denmark A/S, Glostrup, Denmark), and the reactions were visualized by incubating the sections with diaminobenzidine tetrahydrochloride (DAB+) for 5 min. Between incubations the sections were washed with TBS containing 0.5% Triton X-100. Finally, the sections were counterstained with Mayer's haematoxylin.

### Evaluation of TIMP-1 immunohistochemical staining

The study pathologists (MW and RKC) independently scored all the samples which were blinded for clinical data. All the cores represented in the TMA's from a tumour were scored. The vast majority of the cases had identical scoring percentage and intensity of the different cores and in the very few cases that deviated an average was calculated and reported.

The percentage of TIMP-1 positive stained tumour cells was rated as 0 = no cells stained positive, 1 = between >0% and 10% positive, 2 = between >10% and 25%, 3 = between >25% and 50% and 4 = >50% positive cells. Intensity score was made on the basis of the average intensity of staining: 0 = absent, 1 = weak, 2 = moderate and 3 = strong.

The tumour was scored as positive when >25% of the total number of tumour cells stained with a moderate or strong intensity.

### Statistical analyses

The correlation between TIMP-1 expression and clinicopathological parameters was assessed by χ^2 ^statistics and the same applied to the association between TIMP-1 expression and response to chemotherapy.

Overall survival was defined as the elapsed time from date of diagnosis (date of primary surgery) until death attributable to any cause. Univariate overall survival analysis was performed using the Kaplan-Meier estimates and log-rank statistics for comparison of survival curves.

Statistical analyses were performed with the NCSS software (version 2007, Kaysville, Utah, http://www.ncss.com). A value of *p *< 0.05 was considered statistically significant.

## Results

### Patient characteristics and TIMP-1 immunostaining

All patients received first line combination carboplatin and cyclophosphamide as outlined in the protocol [23]. The majority of patients had serous ovarian cancer (79%). The patient characteristics are summarized in Table 1 together with the results of the TIMP-1 immunohistochemical staining.

**Table 1:** Patient characteristics.

Characteristics	No of patients	%	TIMP-1 negative N (%)	TIMP-1 positive N (%)	*P*
**Age**					0.34
<50	47	28.8	44 (93.6)	3 (6.4)	
51-65	94	57.7	80 (85.1)	14 (14.9)	
> 65	22	13.5	19 (86.4)	3 (12.3)	
Median 55.2					
Range 29-70					
					
**FIGO stage**					0.36
I	0	0			
II	26	15.9	25 (96.2)	1 (3.8)	
III	123	75.5	106 (86.2)	17 (13.8)	
IV	14	8.6	12 (85.7)	2 (14.3)	
					
**Tumour grade**					0.32
1	34	20.9	28 (82.4)	6 (17.6)	
2	49	30.1	43 (87.8)	6 (12.2)	
3	66	40.5	61 (92.4)	5 (7.6)	
Not graded (clear cell or metastatic biopsy/cytology only)	14	8.6			
					
**Histopathologic cell type**					0.00004
Serous	129	79.1	119 (92.2)	10 (7.8)	
Endometrioid	11	6.8	9 (81.8)	2 (18.2)	
Clear cell	5	3.1	4 (80.0)	1 (20.0)	
Mucinous	9	5.5	4 (44.4)	5 (55.6)	
Undifferentiated	6	3.7	6 (100)	0 (0.0)	
Carcinosarcoma	3	1.8	1 (33.3)	2 (66.7)	
					
**Residual postoperative tumour**					0.95
≤ 1 cm	60	44.4	53 (88.3)	7 (11.7)	
> 1 cm (Unknown: 28)	75	55.6	66 (88.0)	9 (12.0)	
					
**TIMP immunostaining**					
Negative	143	87.7	NA	NA	NA
Positive	20	12.3			
(<0.1%: 26)					
(0.1-10%: 80)					
(>10-25%: 30)					
(>25-50%:17)					
(>50%: 10)					

Follow up time since date of surgery was at least 15 years with a median follow-up of 1.9 years, (range 0.24-18.1 years) from the date of primary surgery until death or censoring. Survival data were available for all 163 patients. At censoring time (April 2009) 142 patients were deceased (death of any course).

The two senior pathologist' (MW and RKC) independently agreed on the TIMP-1 scoring in 89% of the cases and the interobserver Kappa value was 0.601.

**Figure 2:**
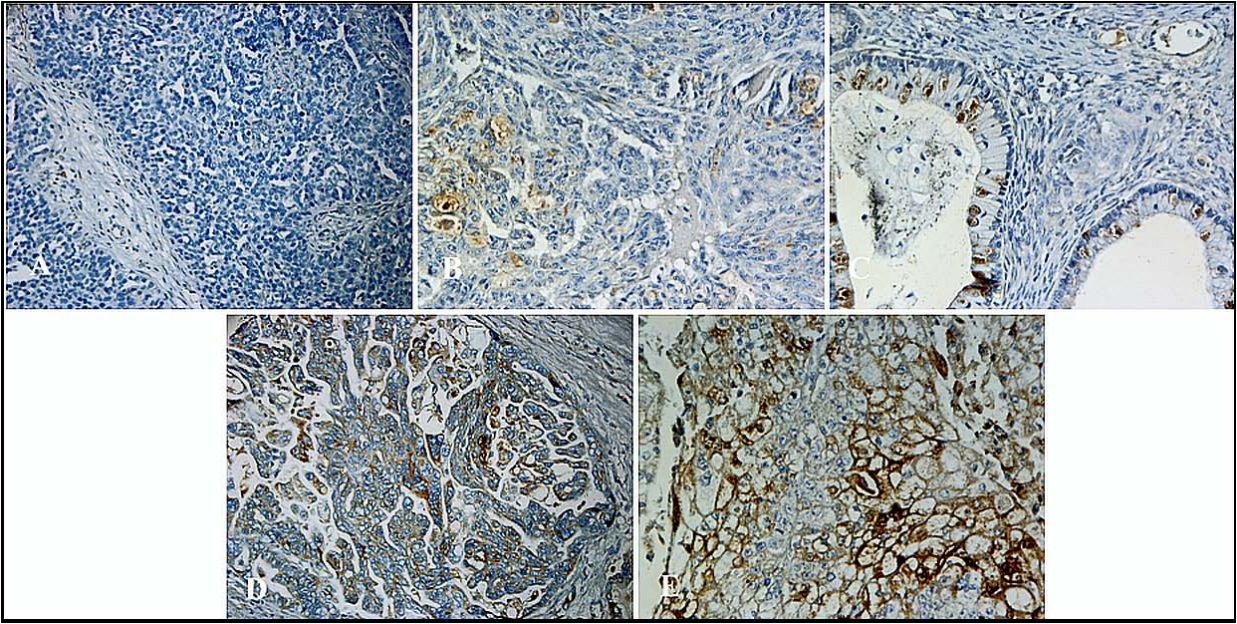
**TIMP-1 immunohistochemical staining**. A. Negative **TIMP-1** staining (X200): Serous adenocarcinoma with staining **intensity=0** (absent) and **percentage** positive cells **=0** (no tumor cells with staining). B. Negative **TIMP-1** staining (X200): Serous adenocarcinoma with staining **intensity=1** (weak) and **percentage** positive cells **=2** (between >10% and 25% tumor cells with staining). C. Negative **TIMP-1** staining (X200): Serous adenocarcinoma with staining **intensity=2** (moderate) and **percentage** positive cells **=1** (between >0% and 10% tumor cells with staining). D. Positive **TIMP-1** staining (X200): Serous adenocarcinoma with staining **intensity=2** (moderate) and **percentage** positive cells **=4** (>50% tumor cells with staining). E. Positive **TIMP-1** staining (X200): Serous adenocarcinoma with staining **intensity=3** (strong) and **percentage** positive cells **=3** (between >25% and 50% tumor cells with staining).

Positive TIMP-1 tumour cell immunoreactivity (Fig. 2) was found in 12.3% of the tumours. Staining was mainly cytoplasmatic although localized membranous staining was also seen. Only scarce staining was found in the stromal compartments. Moreover, immunohistochemical staining with the IgG_1 _monoclonal antibody raised against trinitrophenyl hapten (TNP) did not show any positive staining. TIMP-1 immunoreactivity was not correlated with age at diagnosis, FIGO stage, grade or residual tumour size. Histopathologic cell type was correlated to TIMP-1 immunoreactivity (*p *= 0.00004, χ^2^) with low percentage of TIMP-1 positive cases in serous (7.8%) and undifferentiated tumours (0%) compared to a higher rate of TIMP-1 positive tumours in endometrioid (18.2%, clear cell (20%), mucinous (55.6%) and carcinosarcomas (66.7%). However, these results should be taken with caution since the groups with non-serous tumours were rather small.

The staining of the TMA cores reflected the observation made in twenty whole sections as all patients that scored negative by evaluation of whole sections also scored negative by evaluation of the TMA. The same applied to tumours with positive TIMP-1 expression. In detail, five out of the twenty the whole sections scored positive and the same applied to the five TMA's from the corresponding tumours. None of the fifteen whole sections with negative TIMP-1 score scored positive in the TMA's. Since all cores showed preservation of the original score, tumour heterogeneity did not appear to play an important role.

### TIMP-1 and response

For 106 patients with CA-125 data, the baseline serum CA-125 level (pre-treatment) was elevated to at least twice the upper normal limit. Furthermore, by measuring serum CA-125 at each cycle of chemotherapy it was possible to assess CA-125 response according to GCIG CA-125 response criteria as proposed by Rustin et al [28,29].

Among patients with TIMP-1 negative tumours 87% achieved a CA-125 response compared to patients with TIMP-1 positive tumours where 93% reached a CA-125 response according to GCIG criteria (*p *= 0.53, Table 2). Second look surgery was optional in the protocol and 92 out of the 163 patients had second look surgery performed. For patients with TIMP-1 negative tumours 32% had complete and 52% had partial response by biopsy and clinical assessment at second surgery compared to 18% with complete and 55% with partial response in patients with TIMP-1 positive tumours (*p *= 0.72, Table 3). The total response rate at second look surgery (CR+PR) of 84% for patients with TIMP-1 negative tumours was not significantly different from the 73% total response rate for patients with TIMP-1 positive tumours (*p *= 0.36). Accordingly, TIMP-1 expression status did not seem to influence the response to carboplatin and cyclophosphamide in first line chemotherapy, neither when assessed by CA-125 nor by second look operation.

**Table 2:** Relationship between TIMP-1 tumour cell immunoreactivity and treatment efficacy.

	TIMP-1 expression		
		
	Negative (n = 92)	Positive (n = 14)	*p*
**CA-125 GCIG Response**			0.53
**Response (n = 93)**	80 (87%)	13 (93%)	
**Non-response (n = 13)**	12 (13%)	1 (7%)	

Correlation between TIMP-1 expression and CA125 response (N = 106)

**Table 3:** Relationship between TIMP-1 tumour cell immunoreactivity and treatment efficacy.

	TIMP-1 expression		
		
	Negative (n = 81)	Positive (n = 11)	*p*
**Second look Response**			0.72
**CR** **(n = 28)**	26 (32%)	2 (18%)	
**PR** **(n = 48)**	42 (52%)	6 (55%)	
**SD** **(n = 11)**	9 (11%)	2 (18%)	
**PD** **(n = 5)**	4 (5%)	1 (9%)	

Correlation between TIMP-1 expression and response at second look surgery (N = 92)

### TIMP-1 and survival

The median survival time for all 163 patients was 23.5 months [18.9-27.9 months] 95% CI, with an overall survival range of 3 to 220 months. Significant prognostic markers in univariate survival analysis were age at diagnosis (*p *= 0.004), FIGO stage (*p *= 0.0001), histological tumour grade (*p *= 0.0005), histology (*p *= 0.0005) and residual tumour after primary surgery (*p *= 0.0001). (Data not shown).

**Figure 3:**
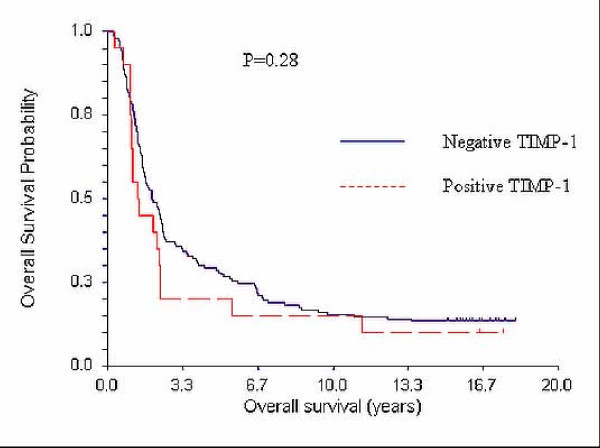
**Kaplan Meier Overall Survival (OS) curves and TIMP-1**. Median OS for patients with TIMP-1 negative tumours (N = 139): 23.7 months [19.0-29.4] 95% CI. Median OS for patients with TIMP-1 positive tumours (N = 21): 15.9 months [12.3-27.4] 95% CI.

The median survival time for the 143 patients with TIMP-1 negative tumours was 23.7 months [19.0-29.4] 95% CI, while the median survival for the 20 patients with TIMP-1 positive tumours was 15.9 months [12.3-27.4] 95% CI. (Fig. 3). Although there seems to be a trend towards longer survival of TIMP-1 negative patients, the results did not show a significant difference (*p *= 0.28, Kaplan Meier, log-rank test).

## Discussion

We demonstrate here that TIMP-1 protein is present in the tumour tissue in a subgroup of ovarian cancer patients but its presence is neither correlated with overall survival nor CA-125 determined response or with objective response to combination chemotherapy with carboplatin and cyclophosphamide.

The monoclonal antibody used for TIMP-1 immunostaining has previously [27] been selected and validated among a panel of anti-TIMP-1 antibodies for its sensitivity and specificity and furthermore, in the present study we found 100% agreement between the scoring results from the TMA and from the whole slides. Moreover, immunohistochemical staining with an IgG_1 _monoclonal antibody raised against trinitrophenyl hapten (TNP) which is a non-sense construct did not show any positive staining. Therefore, we believe that the TIMP-1 immunostaining of the paraffin-embedded slides are correct and robust. We used an arbitrary score of 25% positivity to divide the patients into subgroups as we preferred a simple division into positive and negative. We could also have used a more mathematical approach and multiplied TIMP-1 staining percentage with the staining intensity to achieve an H-score. We actually tried this further subdivision and calculated an H-score but this did not change the final conclusions that TIMP-1 immunoreactivity was not associated with patient outcome in this cohort of ovarian cancer patients. Furthermore, calculating an H-score gave us a score between 0-9 and left us with a rather low number of patients in each group, some of them even very small. This could easily lead to results without scientific value.

In contrast to breast cancer where up to 75% of the tumours appear to be TIMP-1 positive [9] we only scored 12.3% of the cells as TIMP-1 positive. Apart from differences in the assessment of TIMP-1 immunoreactivity this dissimilarity can also be explained by a genuine difference between breast cancer and ovarian cancer. This is underlined by the findings in the present study where TIMP-1 immunoreactivity was significantly lower in patients with serous histology, which constitutes the major part of epithelial ovarian cancers. In breast cancer the most frequent histology is ductal carcinoma. From our data it appears that TIMP-1 immunoreactivity was significantly different for the different histopathologic cell types. The present study showed no worse prognosis in patients with high TIMP-1 expression although no firm conclusion can be made for the group of patients with non-serous cancer since this group were too small for performing a separate survival analysis although it may be interesting to investigate in a larger material whether the higher level of TIMP-1 expression may effect survival in ovarian cancer patients with non-serous tumors.

The previously reported association between high TIMP-1 levels in ovarian cancer patients and shorter survival [18,19] was not observed in our group of patients. However, these two prior studies investigated TIMP-1 levels in serum [18] and plasma [19] in a smaller population including only 59 and 40 ovarian cancer patients, respectively.

In the present study, TIMP-1 expression was detected by means of immunohistochemical staining in TMA made from paraffin blocks of primary ovarian cancer specimens which may be one explanation of the differences compared to studies evaluating levels of TIMP-1 in blood samples. This is supported by studies in primary breast- and colorectal cancer where lack of correlation between tumour tissue TIMP-1 levels and blood levels of TIMP-1 have been published [30,31].

Interestingly, several studies have shown a significantly lower level of TIMP-1 immunohistochemical protein staining in invasive ovarian cancer samples compared to borderline and benign ovarian tumours [32,33], while one study [20] found a higher concentration of TIMP-1 (by ELISA technique in tissue homogenates) in both malignant, borderline and benign tumours compared to the concentration in normal ovaries which underlines the generally increased amount of TIMP-1 protein in ovarian tumours regardless of tumour invasiveness. This is supported by our study in which we did not find statistically higher TIMP-1 immunoreactivity in high grade ovarian cancer patients or in patients with an advanced FIGO stage. In contrast to the studies by Brun et al [33] and by Sakata et al [32], which point to a higher TIMP-1 protein expression in benign and borderline tumours compared to invasive tumours, diagnostic reports have almost all shown higher levels of TIMP-1 in plasma or serum from invasive malignant tumours compared to benign or borderline tumours [17-19]. This discrepancy could be explained by the production of TIMP-1 from other sources than cancer cells, e.g. by stroma cells. Extensive evidence in the literature supports that cancer progression co-depends on the stromal compartment to create a more tumour-promoting microenvironment. In our material the TIMP-1 staining in stroma was rather scarce and the TIMP-1 immunoreactivity in the stroma did not seem to be very pronounced. The study by Brun et al [33] evaluated the TIMP-1 immunoreactivity in stroma and in tumour cells in serous and mucinous ovarian tumours and found a tenfold higher TIMP-1 signal in epithelial tumour cells compared to stroma cells. In a second study by Huang et al [21] of epithelial ovarian tumours also seemed to show lower expression of TIMP-1 in stroma cells than in tumour cells although no direct comparison was made in this study. Consequently, high amounts of TIMP-1 in adjacent tissue do not seem to be an obvious explanation.

By inhibiting MMP activity TIMP-1 is a potential inhibitor of tumour growth and metastasis. It therefore seems paradoxical that elevated expression of TIMP-1 has been associated with more aggressive cancers. However, TIMP-1 may have other functions that are independent of its MMP-inhibiting effect. For example, an antiapoptotic effect and a growth-promoting activity of TIMP-1 on a variety of cell types has been described [2-6] which may partially explain its ambiguous role in tumour progression. However, TIMP-1 in ovarian tumours may not favor tumour growth or metastases explaining the observed lack of association between TIMP-1 and patient prognosis in our study. Nevertheless, it should be emphasized that the lack of correlation between TIMP-1 immunoreactivity and patient outcome may be also explained by other factors such as subsequent additional, different lines of chemotherapy.

Compelling data has been published arguing that TIMP-1 may be associated with response to only certain classes of chemotherapy. In two breast cancer studies lack of tumour cell TIMP-1 immunoreactivity [9] or low levels of TIMP-1 in tumour homogenates [34] predicted sensitivity to antracycline-containing therapy but not to cyclophosphamide, methotrexate and 5-fluorouracil in adjuvant treatment. In another study in which colorectal cancer patients were treated with irinotecan, 5-fluorouracil and folinic acid [10], low plasma TIMP-1 was significantly and independently associated with higher probability of obtaining an objective response to chemotherapy (OR = 3.5, *p *= 0.007). Since antracyclines are topoisomerase-2 inhibitors and irinotecan is a topoisomerase-1 inhibitor TIMP-1 may particularly interact with topoisomerase inhibitors. In the present study patients were treated with carboplatin and cyclophosphamide. Therefore, the lack of an association between TIMP-1 immunoreactivity and response to therapy or survival may be explained by the fact that these cytostatic drugs do not belong to the class of topoisomerase inhibitors, although the evidence for this hypothesis is still limited. The intriguing question raised by these studies of whether TIMP-1 is associated with resistance to topoisomerase inhibitors requires further validation. Ovarian cancer patients are often treated with antracyclines in the case of platinum resistant relapse and it may therefore be of interest to investigate whether positive TIMP-1 immunoreactivity would be associated with resistance to antracycline treatment in platinum resistant ovarian cancer patients.

## Conclusions

TIMP-1 protein is present in the tumour tissue in a subgroup of patients with primary epithelial ovarian cancer. TIMP-1 immunoreactivity was in the present study neither correlated with overall survival nor CA-125 determined response or with objective response to combination chemotherapy with carboplatin and cyclophosphamide.

## Competing interests

The authors declare that they have no competing interests.

## Authors' contributions

KD conceived of the study and carried out design, provision of study patients, data collection, statistical analysis, data interpretation and drafted the manuscript. MW participated in the design of the study, coordination, provision of study material and performed the evaluation of TIMP-1 immunohistochemistry. RKC participated in the evaluation of TIMP-1 immunohistochemistry. AB carried out the TIMP-1 immunohistochemistry and drafted the method section of TIMP-1 immunohistochemical staining. NB participated in the design of the study and in interpretation of data. AJ participated in the design of the study, provision of study patients and in interpretation of data. All authors read and approved the final manuscript.

## Pre-publication history

The pre-publication history for this paper can be accessed here:

http://www.biomedcentral.com/1471-2407/10/185/prepub
